# Supplementary material for: Are carbon emissions trading and green financial instruments synergistic? -Comprehensive quantitative research based on content analysis
Source: PLoS One. 2024 Mar 7;19(3):e0298601. doi: 10.1371/journal.pone.0298601 (PMC10919601; doi:10.1371/journal.pone.0298601)
Supplement: S1 Data — (ZIP) [file pone.0298601.s001.zip › Data/地级市Z_F工作报告.zip!/╣π╢1⁄2/╢1⁄2▌╕╩╨/└┤╫╘╣1⁄2╓┌║┼í╛┬φ┐╦╩2╛▌═°í┐_2007.docx]

各位代表：

　　现在，我代表市人民政府向大会作政府工作报告，请予审议，并请政协各位委员和其他列席人员提出意见。

本届政府工作回顾

　　本届政府任内的三年，是我市经济社会发展再创辉煌的三年。在省委、省政府和市委的坚强领导下，在市人民代表大会及其常务委员会和市政协的监督支持下，我们高举邓小平理论和“三个代表”重要思想伟大旗帜，以科学发展观为指导，深入实施“一城三创五争先”的工作思路，大力推动经济社会双转型，团结奋进，创新创业，全面完成了“十五”计划各项任务，实现了“十一五”的良好开局。

　　——三年来，综合实力在高基数上快速提升。2006年全市生产总值达到2624亿元，比2003年增长72.1%，年均增长19.8%；工业总产值5512亿元，增长88.8%；财政总收入406.5亿元，增长96.9%，其中市财政一般预算收入128.9亿元，增长91.2%；金融机构本外币各项存款余额3464.4亿元，增长54.8%；外贸进出口总额842.2亿美元，其中出口总额473.8亿美元，增长69.2%。三年来，东莞综合实力连续两年位居全国大中城市第十二位，先后获得国家卫生城市、全国绿化模范城市、中国最佳魅力城市、中国最具经济活力城市、中国典范品牌城市、制造业十大最具竞争力城市、中国投资环境百佳城市、国际花园城市等荣誉称号，在国内外的影响力与日俱增。

　　——三年来，城乡建设在高标准上强力推进。城市新区快速崛起。以行政文化中心区为标志的城市新区基本建成，松山湖科技产业园区初具规模，同沙生态区建设全面加快，依山傍水的大城区格局初步形成，各镇街规划建设全面推进，城市的聚集力、辐射力和承载力大幅增强。基础设施全面提升。常虎、龙大、龙林高速和东部快速、环城路一期、松山湖大道、港口大道等一批高等级公路建成通车，市内国道省道和主干道的改造基本完成。虎门港获准建设10个5万吨级深水泊位，其中8个已动工。一批水、电、气等重点工程顺利建成，日供水能力达到600万吨，全年总供电量达到464.5亿千瓦时，东莞大堤和挂影洲围达标工程基本完成，LNG首期竣工启用，固定电话号码成功升至八位，全市信息化综合指数达73%。生态环境明显改善。五大森林公园改造基本完成，全市新增绿地面积80平方公里，建成区绿化覆盖率达41.2%，森林覆盖率达33.1%。全市污水和垃圾日处理能力分别达到42.9万吨和2800吨，市区生活污水和垃圾处理率分别提高到74.8%和100%。新规划的36项污水处理主体工程和主干管网工程全面启动；市财政补偿3.14亿元，关闭水泥厂43家。“四清理”与“五整治”深入实施，城市管理进一步强化。

　　——三年来，增长方式在深层次上逐步转变。产业结构持续优化。省级高新技术企业新增178家，总数达311家，产值占全市的24.8%，提高了4个百分点；高新技术产品出口占全市的34.3%；八大支柱产业的产值占规模以上工业总产值的67.1%；轻重工业比重调整为43∶57；52家市属企业的改制工作顺利完成。全市银行不良资产率三年下降3.22个百分点，金融信贷资产质量居全省第一。拥有国家级展会5个，五星级酒店16家，旅游业经营总收入增长20.1%。内外源经济更加协调。新增个体工商户及私营企业15.8万家，总数达37.5万家，注册资本总额达662.5亿元，增长了87.9%。三年合同利用外资144.1亿美元，实际利用外资111.2亿美元，新增世界500强企业项目11宗，新引进投资总额超1000万美元的外资项目292宗，2006年全市新签外资项目平均合同金额248万美元，比2003年增长1.2倍。一般贸易出口总额增长4.4倍。松山湖和虎门港分别累计引资210亿元和159亿元，东部工业园成为省级工业园区。创新能力有效提升。省、市级民营科技企业分别新增63家和200家，总数分别达到359家和1300家。广东电子工业研究院、华南工业设计院、清洁生产中心等相继成立，新建镇级技术创新平台8个。企业工程技术研究开发中心达56家，其中省级以上14家。新增省级产业集群升级示范区5个。新增省级以上名标名牌产品和国家免检产品240个，总数达305个。2006年全市专利申请量9200件、授权量4300件，均名列全省第四。人才总量达94.6万人，增加35.9万人，建立企业博士后工作站5个。节能降耗稳步推进。清理闲置土地4.1万亩，完成任务总数的91.3%。固定资产投资增幅比2003年回落48.7个百分点，单位生产总值消耗土地下降40.4%、用水下降30.8%、用电下降13%、二氧化硫排放量下降56.9%。

　　——三年来，稳定和谐在多方位上有效体现。安全稳定明显改善。大力整治社会治安，治安形势明显好转，2006年飞车抢夺案比上年下降37.5%，刑事发案数下降20%。安全生产责任落实，全市各类安全生产事故宗数和伤亡人数分别比上年下降6.8%和10.6%，三年来未发生特大安全事故。矛盾调处全面加强，全市信访总量、集体上访批数和人数分别比上年下降19.2%、16.7%和31.7%。民主法制不断完善。全市农村股份合作制改革顺利完成，“村改居”和村（居）民自治稳步推进。政务、村务、厂务公开全面加强，治理商业贿赂专项工作扎实开展。政府新闻发布、政府法律顾问、行政规范性文件管理、行政执法评议考核等制度逐步完善，行政审批电子监察系统首期建成启用，机关作风有效改进。扶贫帮困取得实效。2006年11个欠发达镇生产总值合计379.1亿元，三年增长93.3%，增速比全市快21.2个百分点；81个欠发达村净资产总额20亿元，增长93.3%，资产负债率下降13.4个百分点。城镇居民和农民最低生活保障标准分别提高到每人每月320元和300元。各种社会救助制度进一步健全，困难家庭看病难、住房难和子女读书难有效缓解。三年累计支持对口帮扶地区财物1.93亿元。外来劳动者权益保护进一步加强。企业职工月最低工资标准由450元提高到690元，三年共为50多万人次追回被拖欠工资4.1亿元。

　　——三年来，群众生活在新起点上不断改善。在基本达到宽裕小康的起点上，积极实施民心工程，坚持为群众办实事、办好事，人民群众得到更多实惠。就业更加充分。“创业东莞”工程顺利实施，三年发放就业再就业补贴4657万元，帮助4.2万人成功就业，全市城镇登记失业率控制在3%以下。保障更加健全。基本完成了农（居）民并入职工基本养老保险的改革，实行了计划生育养老保险，全市各项社保参保人数达1075万人次。生活更加宽裕。2004年在全省率先免征农业税。三年来农村村组两级直接和间接分配105.1亿元。2006年，全市城市居民人均可支配收入25320元，比2003年增长37.1%；农民人均纯收入10530元，增长24.2%；城乡居民储蓄存款余额2013.4亿元，增长63.6%。

　　——三年来，社会事业在宽领域上全面进步。教育发展成效明显。免除了全市户籍学生义务教育学杂费；2005年高等教育毛入学率达58.6%，比省定目标提前15年基本普及了高等教育；获得了广东省教育强市称号，27个镇街成为省教育强镇。文化氛围日益浓厚。建成了玉兰大剧院、图书馆、科技馆等一批重点文化设施，新增国家重点文物保护单位3个，总数达到5个；全市现有博物馆21座、图书馆（室）近500个、文化广场447个。在全国率先举办公益文化项目招商，三年共成交项目240个、金额1.77亿元。打造了“文化周末”、“绚丽大舞台”等特色文化品牌。卫生体系逐步健全。完善了市突发公共卫生事件应急机制，全市合法医疗卫生机构达1400个，拥有病床1.4万张，卫生技术人员2万人，实现每个镇街均有1个以上公立综合性医院，每个村（社区）均有1个以上的卫生站。体育事业蓬勃发展。第十二届省运会荣获团体总分第四，成功承办省第七届大学生运动会，莞籍运动员三年获得国际重要赛事金牌16枚、全国赛事金牌63枚，全市人均拥有体育场地面积达到4平方米，群众性体育健身运动广泛开展。

　　三年来，计划生育、民族宗教、外事侨务、工青妇幼、科学普及、档案方志和统计、气象、审计、国防、打私、残疾、老龄等工作也扎实推进，我市实现了全国双拥模范城五连冠，获得了全国文明城市创建工作先进城市称号，社会发展取得了丰硕成果。

　　各位代表！三年来，我们在继承中实现了创新，在发展中凝聚了民心，在奋斗中续写了辉煌。这些成绩的取得，离不开上级党委、政府和市委的正确领导，离不开市人大、市政协与各民主党派的大力支持，离不开广大市民、外来劳动者和投资者以及驻莞部队的共同努力，离不开港澳台同胞、海外侨胞以及国际友人的关心帮助。在此，我代表市政府，向所有参与、支持和关心东莞现代化建设的各界人士，表示衷心的感谢！

　　三年来，我们在推进科学发展与和谐发展的实践中得到了深切的体会：一是必须坚持突出中心与统筹兼顾相统一。既要毫不动摇地突出经济建设这个中心，又要全面发展，统筹安排，协调推进政治、文化和社会建设。二是必须坚持外向带动与自主创新相统一。既要毫不放松地把招商引资作为东莞经济发展的主攻方向，有效保持和巩固外源型经济的优势，又要以外促内，自主发展，形成新的发展动力与优势。三是必须坚持产业升级与城市升级相统一。既要毫不含糊地把推动产业升级作为转型发展的成败关键，又要不失时机地加快城市化步伐，不断增强城市的产业服务功能，使工业化与城市化相互协调，彼此推动。四是必须坚持改革发展与执政为民相统一。既要毫不犹豫地把改革作为发展的内在要求与重要动力，又要牢牢把握改革的方向、力度与方式，充分考虑社会的承受能力，在改革中切实维护好、发展好最广大群众的根本利益。

　　回顾过去的三年，我们也清醒地看到，全市经济社会发展还存在不少矛盾和问题：资源环境压力仍然较大，城乡环境反差明显，水污染问题比较突出；产业结构的优化升级仍需加快，自主创新能力还不够强；各项管理任务繁重，社会治安和消防安全压力较大；区域发展仍不平衡，文明和谐程度有待提高，等等。这些都需要我们高度重视，采取有力措施，在今后的工作中加以解决。

今后五年的目标任务

　　今后五年，政府工作的总体要求和基本思路是：高举邓小平理论和“三个代表”重要思想伟大旗帜，深入落实科学发展观，全面贯彻市第十二次党代会精神，以建设富强和谐新东莞为目标，加快经济社会双转型步伐，全面推进经济、政治、文化、社会建设，努力把东莞打造成现代制造业名城、创新创业热土、宜居生态城市、和谐幸福家园，率先基本实现社会主义现代化，争当落实科学发展、构建和谐社会与加强党的建设的排头兵。

　　今后五年，我市经济社会发展的主要预期目标是：全市生产总值年均增长13%以上，争取五年翻一番；人均GDP达到7.8万元；三大产业增加值比重调整为1:52:47；进出口总额年均增长10%；单位生产总值能耗年均下降4.4%，2010年比“十五”期末降低20%；全市资源综合利用率达到40%；城市化水平达到80%；城市信息化综合指数达到80%；环境综合指标达到90分；城镇登记失业率控制在3%以内，率先基本实现社会主义现代化。

　　一、经济发展转入新轨道

　　推动经济发展从资源依赖、投资驱动和粗放增长，转入创新主导、技术支撑与集约发展的新轨道。

　　加快推进结构调整。做强制造业，推动制造业的规模化、高级化、信息化和适度重型化。加快推进松山湖、虎门港、东部工业园和东部快速路两旁土地的开发建设。努力引进高新技术产业、龙头企业和研发型企业，力争高新技术产业增加值占工业增加值的38%。重点扶持电子信息、电气机械等八大支柱产业。做大服务业，大力发展现代商贸、物流、会展、旅游等产业以及法律、设计、中介、会计、咨询等专业服务，推进金融创新与资源整合，建设金融强市。做优大农业，积极发展现代化都市型农业，努力实现产业特色化、生产标准化、设施现代化，2010年全市建成10万亩以上的标准化农田。

　　大力加强自主创新。以政府投入为引导，企业投入为主体，政策扶持为保障，着力构建科技投融资机制。以松山湖、专业镇和产业集群为依托，建设一批公共技术平台和行业技术平台。以高新技术企业、研发机构和新兴服务业为载体，大力引进和造就高素质人才，培育和弘扬创新文化。以知识产权保护和产学研合作为手段，鼓励企业研发自主技术，创建自有品牌，力争到2011年再创10个中国名牌产品、50个国家免检产品和100个省名牌产品。

　　严格实施节能降耗。将节能降耗指标纳入全市经济社会发展综合评价和年度考核，明确重点行业、企业节能降耗的目标责任和相关部门的监管责任。积极推广节能降耗与资源综合利用的技术、工艺和设备。从项目审批、资源供给、市场准入等方面，有效推进节能降耗工作的开展。

　　二、城乡环境增创新优势

　　实施“优化、整合、提升”战略，继续加快城市建设步伐，增创城乡环境新优势。

　　优化功能布局。加大市区、同沙、松山湖与东部快速两侧统筹带“四位一体”的整合力度；以东莞大道为轴线，强化中央商务区功能；以东纵大道、东城大道、胜和片区、西正街、华南MALL为重点，强化商贸中心功能；以中央生活区、成熟社区建设为重点，强化城市居住功能。加强对各组团城镇的指导与整合，实现统筹规划、功能互补。

　　优化基础设施。进一步完善现代化交通体系。完善铁路沿线站场布局，加快市域轻轨报批和建设，配合推进广深港客运专线和广珠城际轨道等项目；力争建成广深沿江高速、博深高速、莞深高速三期和环莞快速路等；加快镇、村两级联网道路升级改造。虎门港建成15个5万吨级以上的深水泊位，新增吞吐能力4000万吨。大力完善全市供排水、供电、供气、防灾减灾、信息等重要基础设施网络，完成东江与水库联网供水工程。

　　优化生态环境。深入实施农村环境“五整治”，2010年全面完成整治任务，构筑城乡一体化的优美环境。加快环保基础设施建设，加强污染治理，力争全市生活污水处理率达90%以上，生活垃圾无害化集中处置率100%，工业废水达标排放率100%。以“护山、净水、保农田”为重点，构筑“三河两网一通道”的区域生态格局，加强饮用水水源保护，每年改造水源涵养林1600公顷，建立海洋保护区。

　　优化城市管理。完善规划体系，强化规划实施，确保规划的先导性和权威性。理顺城市管理体制，健全基层管理机构，强化社区公共管理和社会服务功能。2010年全市80%以上的社区成为“六好”平安和谐社区。加强城市管理综合执法，形成规范化、精细化、信息化的城市管理长效机制。

　　三、社会和谐构建新格局

　　着力维护社会稳定，有效促进区域协调，切实保障成果共享，努力构建社会和谐新格局。

　　在综合治理中维护和谐。实施科技强警，全市设置10万个治安视频监控点，推动视频监控进路街、进社区、进公交、进出租屋；完善治安防范体系，建设现代警务机制，提高快速反应和联防联动能力；保持严打高压态势，建立群防群治长效机制，实现社会治安根本性好转。严格落实安全生产责任制，全面排除安全隐患，进一步健全社会预警体系和应急处置机制，提高保障公共安全和处置突发事件的能力。完善矛盾纠纷排查调处机制，从源头上减少不稳定因素。

　　在统筹发展中体现和谐。统筹城乡发展，大力推进公共财政向农村倾斜，建设重点向农村推进，公共服务向农村延伸，实现城乡服务均等、一体发展、共同繁荣。统筹区域发展，加大对欠发达镇村的扶持力度，有效改善发展环境，增强发展后劲。

　　在强化保障中促进和谐。建立城乡一体、全面覆盖、服务完善的社会保障和救助体系。加强妇女儿童权益保护，完善老年人优待措施，推进残疾人康复、教育和就业。大力实施“新东莞人”工程，积极开展“城市暖流”等活动，切实保障劳动者权益，大力营造互敬互爱、公正公平、共享成果、共建和谐的良好氛围。

　　四、民主法制取得新进展

　　积极发展民主政治，规范政府职能，推进依法治市，建设社会主义政治文明。

　　扩大民主参与。坚持民主决策，健全广泛调研、公众参与、专家论证和政府决定相结合的行政决策机制。扩大政务公开，强化民主监督。健全基层民主，完善管理制度，切实保障基层群众的知情权、参与权和监督权。

　　转变政府职能。改善经济调节，强化市场监管，更加注重社会管理和公共服务。落实《行政许可法》的规定，全面清理审批事项和部门规章，消除行政垄断、玩忽职守等越位、失位行为。推进管理创新，依法简政放权，完善电子政务，提高行政效能，改进机关作风，努力建设服务型政府。

　　推进依法行政。完善政府立规，提高政府运用法律手段管理经济和社会事务的能力。严格照章办事，依规管理，建立权责明确、行为规范、监督有效、保障有力的执法体制。推进廉政建设，完善源头防范、权力制约和内部监督等机制，实现有权必有责、用权受监督、违法受追究。

　　五、富裕文明迈上新台阶

　　落实富民惠民措施，大力改善群众生活，提高群众的幸福指数，实现人的全面发展与社会全面进步。

　　努力实现共同富裕。全面实施“创业东莞”工程，用好10亿元的专项资金，完善就业创业的鼓励政策与服务体系，促进全民就业创业；加强农村集体资产管理，进一步壮大集体经济，稳步增加农民收入。发展社会福利和慈善事业。

　　大力弘扬现代文明。高水平发展教育事业，全市户籍人口高中教育毛入学率达98%，高等教育毛入学率达60%以上，基本实现教育现代化。全面推进图书馆之城、博物馆之城、广场文化之城建设，构建公共文化服务体系，深化文化体制改革，繁荣城乡文化。积极开展现代公民教育和思想道德建设，广泛倡导“八荣八耻”的社会主义荣辱观，大力弘扬“海纳百川、厚德务实”的东莞城市精神，全面提升市民的现代文明素质，争取2008年创建为全国文明城市。

　　积极打造健康城市。完善城乡医疗卫生设施，深化医疗卫生体制改革，整顿医疗卫生管理秩序，优化公共医疗卫生服务，努力实现人人享有优质卫生保健。积极发展群众体育、竞技体育与体育产业。合理控制人口总量，优化人口结构，提高人口综合素质。

　　2007年工作安排

　　2007年是我市承前启后、转型发展的重要一年。今年经济社会发展的主要目标是：生产总值增长15%；单位生产总值能耗下降4.4%；工业总产值增长18%；市财政一般预算收入增长15%；固定资产投资总额增长20%；社会消费品零售总额增长14%；外贸出口总额增长12%；实际利用外资增长12%；居民消费价格指数上升控制在3个百分点以内；城市居民人均可支配收入增长9%；农民人均纯收入增长7%；人口自然增长率控制在7‰以内。

　　今年政府工作的指导思想是：坚持以科学发展、和谐发展为主线，认真落实市第十二次党代会的各项部署，抓住机遇，乘势而上，积极推进经济社会双转型，大力提升安定和谐的社会形象、创新活力的经济形象、环境优美的城市形象、文明崇德的素质形象，努力实现又好又快发展。具体要抓好以下十个方面的工作：

　　一、维护安全稳定，建设平安东莞

　　坚持把安全稳定作为社会管理的突出任务，毫不松懈地抓紧抓实。

　　着力维护社会治安。建成科技强警示范市，深入推进警务机制改革，严厉打击“两抢一盗”等多发性犯罪，加大对入室和公交车上作案的打击力度，大力整治“黑网吧”，奖励见义勇为与举报犯罪行为。巩固扩大“治摩”成果，无异地摩托车村（社区）达到95%以上；9月1日起分步实施禁止本地摩托车在市内行驶。落实综治责任，创新管理办法，强化出租屋管理，从源头上消除各种治安隐患，确保治安状况持续好转。

　　着力落实安全生产。进一步健全监管网络，大力加强执法监管。深入开展危险化学品、水陆交通、“三合一”工厂、“三小”场所等专项整治，全面开展公路桥梁、旧厂房的检测，防范和遏制重特大安全事故的发生。加强自然灾害预报预测，健全社会预警体系和应急处理机制。坚决整治“黑诊所”和各类非法行医行为，基本清除非法行医窝点；强化日常监管，突出重点整治，确保食品药品安全。

　　着力强化矛盾调处。进一步完善市、镇、村、企业四级信访调解网络，充分发挥工、青、妇等群团组织以及民族、宗教工作机构的作用，有效整合基层综治力量，积极构建“大调解”格局。密切注视社会动态，防范各种社会冲突事件发生。落实信访工作属地负责、领导包案制度，加大普法教育、劳动监察、法律援助等力度，妥善处理劳资纠纷、征地拆迁等引发的矛盾，做到发现在源头，控制在萌芽，化解在基层。

　　二、加强招商引资，巩固外源优势

　　坚持把招商引资作为经济发展的主攻方向，进一步巩固我市外源型经济的优势和特色。

　　有效整合招商引资力量。加强市、镇外经贸部门工作力量，明确工作职责，形成工作合力；健全招商引资工作领导协调机制，提高项目审批“一条龙”服务质量，完善外商联络小组协调会、集中服务日、世界500强企业跟踪服务、加工贸易联网监管等制度；实施招商激励机制，表彰和奖励招商引资有功单位和人员。

　　有效整合产业发展载体。突出加快市级三大园区建设，实施大项目带动、大平台支撑的战略，提高吸引外资的质量和水平，尽快扩大产业规模。松山湖积极申报国家级园区，重点推进北部工业城和配套设施建设，积极促进已签项目早日投产；虎门港建成启用第一批2个深水泊位，加快沙田港区一、二期工程建设，抓紧国家保税物流中心的立项报批，完善配套设施，有效承接临港工业与现代物流业；东部工业园加大开发建设统筹力度，尽快完善基础设施；东部快速路两旁统筹土地抓紧规划编制工作。各镇街抓好原有工业园区和工业用地的整合，实施拆旧建新、拆小建大、拆零建园，完善设施，优化布局，提高管理水平和承接产业能力。

　　有效整合外资企业资源。更加注重以商引商，加强与外商协会及重点企业的联系，改善营商环境，鼓励在莞外资企业增资扩产，并通过他们加强对外推介工作。更加注重产业招商，依托东莞的产业基础，发挥产业集聚优势，吸引配套企业前来投资。

　　有效整合招商引资宣传。对内做好思想发动，统一宣传口径，正确面对困难，增强招商信心。对外拓宽沟通渠道，更多地邀请跨国企业和外国商会组织来莞参观考察，选择欧美日韩地区重点城市、重点项目进行有针对性的宣传推介。

　　三、加快自主创新，增强发展能力

　　坚持以引进消化吸收再创新为基本模式，大力实施自主创新战略，有效增强发展后劲。

　　强化政府投入的引导作用。用好市财政每年10亿元的“科技东莞”专项资金，抓紧完善相关操作办法与配套措施。加快建立科技创业投资引导基金，推动政策性银行、商业银行与社会资本构筑中小企业融资平台。鼓励企业加大技改投入，推动社会投资科技创新项目。

　　强化“两自”企业的主体作用。完善专利服务与知识产权保护。支持企业承担粤港关键领域重点突破项目、省技术进步项目以及参与制订国际、国家和行业技术标准。重点扶持全市装备制造业20强企业和民营50强企业，培育一批自主创新型龙头企业。积极扶持中小科技企业。引导和支持相关企业争创国家和省名牌、免检产品和驰名、著名商标，不断扩大名优产品的行业覆盖面和市场占有率。

　　强化创新平台的载体作用。抓好公共技术平台、行业技术平台、企业研发机构的建设，办好东莞国际科技合作周等科技交流活动。积极筹建东莞华中科技大学制造工程研究院、莞港生产力促进基地等重大项目，完善东莞市毛纺织技术创新中心、国家模具重点实验室东莞实验中心等行业创新平台。

　　强化人才队伍的支撑作用。制定覆盖面更广的人才户籍准入制度，完善人才服务与配套政策，充分发挥留学人员创业园、博士创业园、中小企业创业园的承载与集聚作用，依托创新项目与优质企业，积极引进更多的高素质人才，为自主创新提供强大的智力支撑。

　　四、推进五项整治，优化城乡环境

　　坚持把环境整治作为城乡建设的有力抓手，深入推进“五整治”，努力营造城乡一体化的优美环境。

　　全面完成环境卫生整治和农贸市场整治。按照“国家卫生城市”的标准，全面落实12项内容63个专项整治任务，实现卫生保洁全覆盖。继续推进农贸市场的后续整治工作，重点抓好不合格市场的整治，巩固现有整治成果。完善长效管理机制，大力整治乱停乱放、乱摆卖、乱拉挂等现象。6月底前全面完成上述整治。

　　全面推进旧村整治和生态环境整治。加大旧村改造力度，稳步建设农民公寓，完成全市45个试点村整治，每个镇街至少有25—30%的村铺开整治。加快生态建设工程实施步伐，实现“三个60”的目标：市属26项重点生态建设工程完成60%的工程量；镇属615项生态工程完成60%的工程量；全市新增绿地60平方公里。

　　全面加快城市基础设施和功能配套建设。推进路网工程建设，完成西部干道、滨江西路、广园东快速连接线、松山湖兴园路—同沙环湖路连接线、环城路—广深高速石鼓连接线以及镇街联网公路升级改造；抓紧环城路北环段、环莞快速路一期、莞深高速三期、惠常高速东莞段、常虎高速新联支线、环城路—广深高速望牛墩连接线建设；抓紧轨道交通和沿线站点规划建设的前期筹备工作，启动东莞火车东站改扩建工程，实施东江航道整治。加快市人民医院新院、市第三人民医院新院、市中医院新院、市妇幼保健院新院和市疾病预防控制中心“四院一中心”的规划建设，总投资约25亿元。建成市第六水厂一期。全面完成小型水库达标建设，基本建成220个城乡水利防灾减灾工程项目。建设110千伏及以上输变电工程44项，总投资18亿元，力争年内投产25项，新增主变容量455.5万千伏安。加快LNG管网建设。

　　五、落实扶持政策，壮大民营经济

　　坚持把民营经济作为自主发展的重要力量，全面落实扶持政策，不断提高竞争能力。

　　突出政策落实。加强部门协调和工作督查，完善实施细则与操作办法，着力落实民营经济“新48条”，在前置审批、用地用工、人才引进、政府采购等方面，给予民营企业更多的方便和支持。用好民营企业技术改造、创业发展和科技兴贸等专项扶持资金。放宽投资领域，促进公平竞争，完善中小企业的社会化服务体系。

　　突出扶优扶强。实施大企业培育战略，选择一批规模较大、管理先进的民营龙头企业，通过上市、并购、重组等途径，优化资源配置，增强竞争优势，不断做大做强。积极发展产业集群，每年安排专项资金，推进产业集群的公共平台建设，为技术研发和推广提供良好支撑。

　　突出内外融合。大力推动外资企业与民营企业合资合作，继续完善民营企业与外资企业配套产品目录交流机制，引导民营企业更多、更深入地进入外资企业的产业链。鼓励民营企业扩大出口、向外拓展。

　　六、实施五大工程，推动环保节约

　　坚持把环保节约作为持续发展的重要保障，积极创建国家环保模范城市、国家园林城市和生态林业市，促进人与自然的和谐发展。

　　大力实施碧水工程。突出“治水为先”。力争实现城乡污水处理全覆盖，全市日污水处理规模达到251.4万吨；强化重点污染企业整治，对全市1252家重点污染企业，分类实施关闭、搬迁和在线监控；投入4.5亿元，对全市285公里的主要内河涌进行清淤疏浚、修建堤岸和两岸绿化。

　　大力实施蓝天工程。完成沙角电厂群脱硫工程、市域内各电厂热电联产机组、大型工业锅炉的烟尘净化装置技改建设；实行机动车尾气排放国III标准，推广使用无铅汽油；积极推进饮食业“油改气”，引导公交车和出租车“气代油”，大力推广使用天然气，提高清洁能源使用率。

　　大力实施绿地工程。以山、水、田、林等为重点，划定1179平方公里的城市生态绿线控制范围，保护好现有生态环境。以增加绿量、提高绿质为目标，建设山上绿林、田园绿片、环城绿带、广场绿地和小区绿景，构建沿路、沿江、沿河绿化走廊，形成网络化、多层次的绿化格局，改善城市绿化景观和生态功能。

　　大力实施宜居工程。建成日处理规模20吨的市医疗废物集中焚烧处理中心，实现医疗废物100%集中处理；建成日处理规模1000吨的潲水处理厂；加快建设污泥处理工程和山区片、水乡片、沿海片、长安镇等4项垃圾处理工程。加强日常监督管理，减少交通、建筑施工和生活噪声污染，营造清洁宁静的宜居环境。

　　大力实施绿色GDP工程。着力推行节能降耗，固定资产投资项目立项增加节能评估审查，对列入省“双千节能行动”的70家重点耗能企业实施强制能耗审计与技术改造，在建筑领域实施节能示范工程。着力推行清洁生产，推广应用再生能源，发展循环经济。着力推行集约用地，按照“严控总量，盘活存量，提高质量”的原则，进一步提高市镇两级统筹调控土地的能力，严格实行依法用地。

　　七、扶持镇村发展，促进区域协调

　　坚持把扶持镇村作为区域协调的有效途径，统筹城乡发展，以工促农，以城带乡，促进共同富裕。

　　加大财政扶持力度。建立镇街工商税收超收奖励的财政激励机制，并适当向经济欠发达镇倾斜。从今年开始，原由村承担的教育经费、最低生活保障金等改由市、镇两级分担，减轻村级负担。落实市、镇两级专项资金，重点完善农村道路、供水等设施，深入推进“五有”新村建设，进一步改善农村生产生活条件。

　　加快发展都市型农业。今年起连续3年安排专项资金，以贴息贷款的方式，培育和壮大一批市级农业龙头企业。落实1亿元专项资金，抓紧建设首批6个市级农业产业园，启动第二批4个的规划建设。实施科技兴农战略，建立一批科技示范基地，完善农业科技服务和信息服务，推动农民专业合作组织发展。

　　加速欠发达镇村发展。继续向11个欠发达镇提供每镇1亿元的扶贫贴息贷款。今年起连续三年，对每个欠发达村500万元以内的贷款提供贴息，帮助解决启动资金问题。继续向欠发达镇村返还土地有偿使用费、土地出让金市留成部分等，进一步完善和落实对口帮扶。

　　加强农村集体资产管理。出台新的农村集体资产管理实施办法，完善农村股份合作制改革的后续管理，规范股份合作经济组织运作。加强集体土地征用补偿和集体建设用地使用权流转收益的管理，抓好集体资产和财务管理“八镇百村”示范工程，全面开展村级干部任期经济责任审计。

　　八、发展社会事业，提高文明程度

　　坚持经济社会统筹发展，以创建全国文明城市为契机，推动各项社会事业全面进步。

　　进一步增强文化活力。继续举办读书节、学习论坛、走进东莞文明等活动，建设学习型城市。组建岭南画院和文学艺术院，加快文艺创作基地建设，启动文艺精品创作工程，丰富和活跃广场文化、社区文化与企业文化。加强文物保护和非物质文化遗产保护。深化文化事业管理体制改革，推进文化市场综合执法。整合全市广电网络，实施有线电视数字化整体平移工程。

　　进一步提升教育水平。市财政增加投入8.4亿元，推动全市由三级办学向二级办学转变。推进规范化学校建设，力争全市义务教育规范化学校达80%。积极推进高中联合办学、规模化办学，加快示范性普通高中的建设。启动职业高中联合办学与市职业技术学院建设，加快职业教育重点专业与实训基地建设。充分发挥高等教育的地方特色、服务功能与创新活力。加强师资队伍建设，提高教学科研水平。力争全市所有镇街都成为省教育强镇。

　　进一步发展卫生体育社保等事业。深入推进卫生综合改革，完善医疗管理体制，增强社区医疗卫生服务。抓好群众体育健身设施建设，开展全民健身运动，进一步发展竞技体育和体育产业。提高农民医疗保险待遇水平，推进职工基本养老保险制度改革，建立地方养老保险制度，适度推进社保扩面征缴。积极争创“全国双拥模范城”六连冠。推进计划生育“三项工程”，提高出生人口素质。继续做好民族、宗教、外事、侨务、台务、统计、人防、档案、方志等工作。

　　九、办好十件实事，增进社会和谐

　　坚持把改善民生作为构建和谐的基础工程，继续高度关注与民生密切相关的热点、难点问题，集中力量为群众办好十件实事：一是加强社会治安。全市建成500个社区警务室，建设社区治安视频监控点5000个；在主要路口、干线出入口装设卡口系统150套；为1200多辆警车安装GPS卫星定位系统。二是确保食品安全。学校和企业的集体食堂监管覆盖率分别达到100%和80%；基本完成东莞市产品质量检测基地一期建设；建立供莞水产品定点生产基地3000亩；生猪定点屠宰率达90%。三是优化社区医疗服务。市镇加大公共财政补贴力度，加强社区卫生服务和基层医疗网点建设，确保社区居民步行15分钟内能就近获得基本医疗预防保健服务。四是完善公共交通。落实公交优先，全市新增公交车3056辆；在市四条主干公路沿线改造、新建公交候车亭260座。五是加快污水治理。建成32座污水处理厂的主体工程，完成全市截污主干管网建设。六是改善农村环境卫生。建设公共厕所1500座，垃圾压缩站1100个，整改垃圾填埋场166个。七是减轻教育负担。从9月起，免收户籍学生义务教育阶段书本费，深入治理中小学教育乱收费行为，规范民办学校收费。八是实施通路通水工程。市镇两级共投入2.1亿元，帮助393个自然村通水泥公路、104个自然村接通自来水，全面解决农村“行路难”和“饮水难”问题。九是推进充分就业和残疾人帮扶。培训户籍人员8000人，新增城镇就业岗位3.8万个，免费推荐就业1.8万人次，实现户籍大中专毕业生充分就业，退役士兵职业技能培训参训率达85%以上；为19000名一至四级户籍困难残疾人提供生活补助，为9500名户籍残疾人提供医疗康复、教育培训和就业服务。十是保护外来员工合法权益。镇村劳动争议调解率达到90%以上，纳入工资监控的企业1.8万家，“12333”劳动社保信访平台提供24小时服务。

　　十、坚持依法行政，提升施政水平

　　坚持以提升政府系统的思想力、执行力、协同力、公信力、亲和力、自省力为目标，全面推进依法行政。

　　以政府立规促进规范行政。推进政府立规工作，督促部门和镇街执行规范性文件的事前审查和事后备案，进一步完善和落实规范性文件定期清理和修改废止制度。健全政府法律顾问、专家咨询论证、立规征求意见和立规听证等制度，有效扩大公众参与程度。

　　以公平合理促进公正行政。坚持公正合理、科学规范的原则，加快完善行政程序，严格遵守法定职权，严肃履行法定程序，在行政过程中做到不徇私、不歧视、公道处事、合理裁量。做好行政复议工作，对个别部门、个别人员有章不循、程序失当、显失公平的，严厉查处，及时纠正。

　　以强化监督促进阳光行政。依法扩大政务公开范围，主动接受人大监督、政协监督、社会监督，强化政府内部监督。全面完成行政审批电子监察系统二、三期建设。强化财政资金的使用监督和绩效评价，积极推进效益审计，加强市属企业和资产的监管。坚决惩治以权谋私、商业贿赂等腐败行为。畅通投诉渠道，继续开展行风评议和“市民评机关”活动。

　　以素质提高促进文明行政。落实机关工作人员法律知识学习制度、依法行政情况考核制度；落实行政执法人员《行政许可法》等五法培训制度，经考核后持证上岗。加强公务员能力建设和职业道德教育，积极改进机关作风，增强服务意识和责任意识，坚决整治不作为和乱作为。

　　以管理创新促进高效行政。积极推进行政审批制度改革，清理审批事项，简化办事程序，实行简政放权。发挥市行政服务中心作用，完善“窗口服务”，推行电子政务、“一站式办公”和“网上并联审批”，切实解决群众和企业“办事难”问题。

　　各位代表！推进经济社会双转型、建设富强和谐新东莞的宏伟蓝图已经绘就，政府的使命光荣而艰巨。让我们同心同德，奋发前进，深入落实科学发展，努力构建和谐社会，为东莞率先基本实现现代化而努力奋斗！
